# Relationship between staple food types and cardiovascular disease risk among older Chinese adults

**DOI:** 10.3389/fnut.2025.1539920

**Published:** 2025-05-22

**Authors:** Chunmei Chen, Fayun Zhao, Maozong Du, Xin Wang

**Affiliations:** Department of Cardiology, Henan Provincial Chest Hospital, Zhengzhou University, Zhengzhou, China

**Keywords:** cardiovascular disease, staple food, rice, wheat, older adults

## Abstract

**Background:**

Dietary habits, particularly staple food consumption, play a significant role in influencing cardiovascular disease (CVD) risk. However, limited research has examined the relationship between staple food types and CVD incidence in aging populations, especially in China. This study aims to identify which types of staple foods are most beneficial for cardiovascular health among older Chinese adults.

**Methods:**

Data from the Chinese Longitudinal Healthy Longevity Survey (CLHLS) were analyzed to explore the associations between staple food types (rice, wheat, and coarse cereals) and CVD risk among 16,498 adults aged 65 and older. Cox proportional hazards models were employed to evaluate the relationship between staple food types and CVD incidence, while restricted cubic splines assessed potential non-linear relationships between staple food intake and CVD risk. Stratified analyses were performed based on age, sex, and hypertension history.

**Results:**

During a median follow-up of 7.38 years, 1757 participants experienced new-onset CVD. Wheat as a staple food was related to a 40.8% higher risk of CVD compared to rice (HR: 1.408; 95% CI: 1.195–1.658; *p* < 0.001), while no significant association was observed for coarse cereals. Stratified analyses revealed that the association with wheat was stronger among participants aged 65–79 years, males, and those without hypertension. No linear relationship was found between intake levels of rice, wheat, or coarse cereals and CVD risk, but non-linear associations emerged for rice and wheat intake (P for non-linear association<0.001 and = 0.010, respectively). A U-shaped relationship was observed for wheat, with the lowest CVD risk at a cooked intake of 375 g/day, consistent with dietary guidelines.

**Conclusion:**

This study highlights the differential impact of staple food types on CVD risk, with wheat consumption linked to a higher incidence of CVD compared to rice, particularly in specific subgroups. These findings provide evidence to inform dietary guidelines for older Chinese adults and underscore the need for further research into the underlying mechanisms.

## 1 Introduction

Cardiovascular disease (CVD) is the primary cause of death and disability globally, presenting substantial challenges for public health and healthcare systems (1–3). With the global population aging rapidly, the burden of CVD is particularly pronounced among older adults. It accounts for approximately 24% of disability-adjusted life years (DALYs) in individuals aged 65 and older and remains the leading cause of death in this age group (4). In China, where population aging is accelerating, CVD is a primary health threat for both urban and rural residents. As of 2021, CVD accounted for 48.98% of deaths in rural areas and 47.35% in urban areas, with two out of every five deaths attributed to the disease (5). Identifying factors that contribute to CVD risk is therefore critical for early prevention and intervention.

Prevention is the most effective and economical approach to reducing the burden of CVD. Among modifiable risk factors, diet plays a pivotal role in influencing CVD outcomes (6–8). Grains, as a major component of diets worldwide, contribute 56% of global energy intake and 50% of protein intake, forming the foundation of recommended daily dietary guidelines (9). Emerging evidence, supported by systematic reviews and meta-analyses, suggests that whole grain cereals may protect against CVD (8, 10–12). In China, dietary patterns differ significantly from those in Western countries, with white rice and wheat flour products, such as steamed buns and noodles, being the primary staple foods. Previous studies have primarily focused on the consumption of individual staple foods, but their findings have been inconsistent. For example, a study based on the China Health and Nutrition Survey reported that high rice intake was associated with a 50% reduction in CVD risk, whereas high wheat intake was linked to a twofold increase in CVD risk (13). However, systematic review and meta-analysis found no significant association between white rice consumption and CVD incidence (14, 15). Additionally, two studies conducted in U.S. populations reported no association between rice consumption and either subclinical CVD markers or overall CVD risk (16, 17). Notably, recent study has highlighted that China has the highest age-standardized rates of diet-related CVD deaths worldwide, underscoring the critical need to understand the dietary contributions to CVD risk in this context (18). However, existing studies have largely examined the effects of single staple foods rather than directly comparing different staple food types. Limited research has explored the association between specific types of staple foods, such as white rice and wheat flour products, and CVD risk. Identifying which staple foods may be more beneficial for CVD prevention remains a critical knowledge gap.

This study utilizes data from the Chinese Longitudinal Healthy Longevity Survey (CLHLS) to investigate the relationship between staple food types and CVD incidence among Chinese older adults. It examines the strength and nature of dose–response associations between the intake of specific staple foods and CVD risk. A clearer understanding of how staple food choices impact CVD risk can inform public health strategies and dietary recommendations tailored to the Chinese population.

## 2 Materials and methods

### 2.1 Data sources and study population

This study utilized data from the CLHLS, a nationwide, ongoing longitudinal study initiated in 1998. The most recent data available are from the 2018 wave. A multistage, stratified cluster sampling method was used to create study population from 631 cities and counties across 22 of China’s 31 provinces. The study focused on community-dwelling older adults, with an emphasis on oversampling the oldest-old population. To account for the high mortality rate among the oldest-old participants, replacements of deceased and lost-to-follow-up individuals were recruited in each wave. Loss to follow-up ranged from 8.3 to 20.6% between survey years (19). Sampling weights were calculated to ensure that the findings could be generalized to the national population of older adults. These weights accounted for variations by age, sex, and rural or urban residence. For instance, the weight variable for the 1998 survey wave was derived using population estimates from the 2000 census for the 22 provinces included in that wave. These estimates were based on full census data and adjusted to reflect the demographic distribution of the oldest old population at that time. The use of sampling weights allowed for nationally representative analysis and helped correct for potential sampling biases. A detailed explanation of the study design and sampling procedures can be found in previously published methodological reports (20, 21).

**Figure 1:**
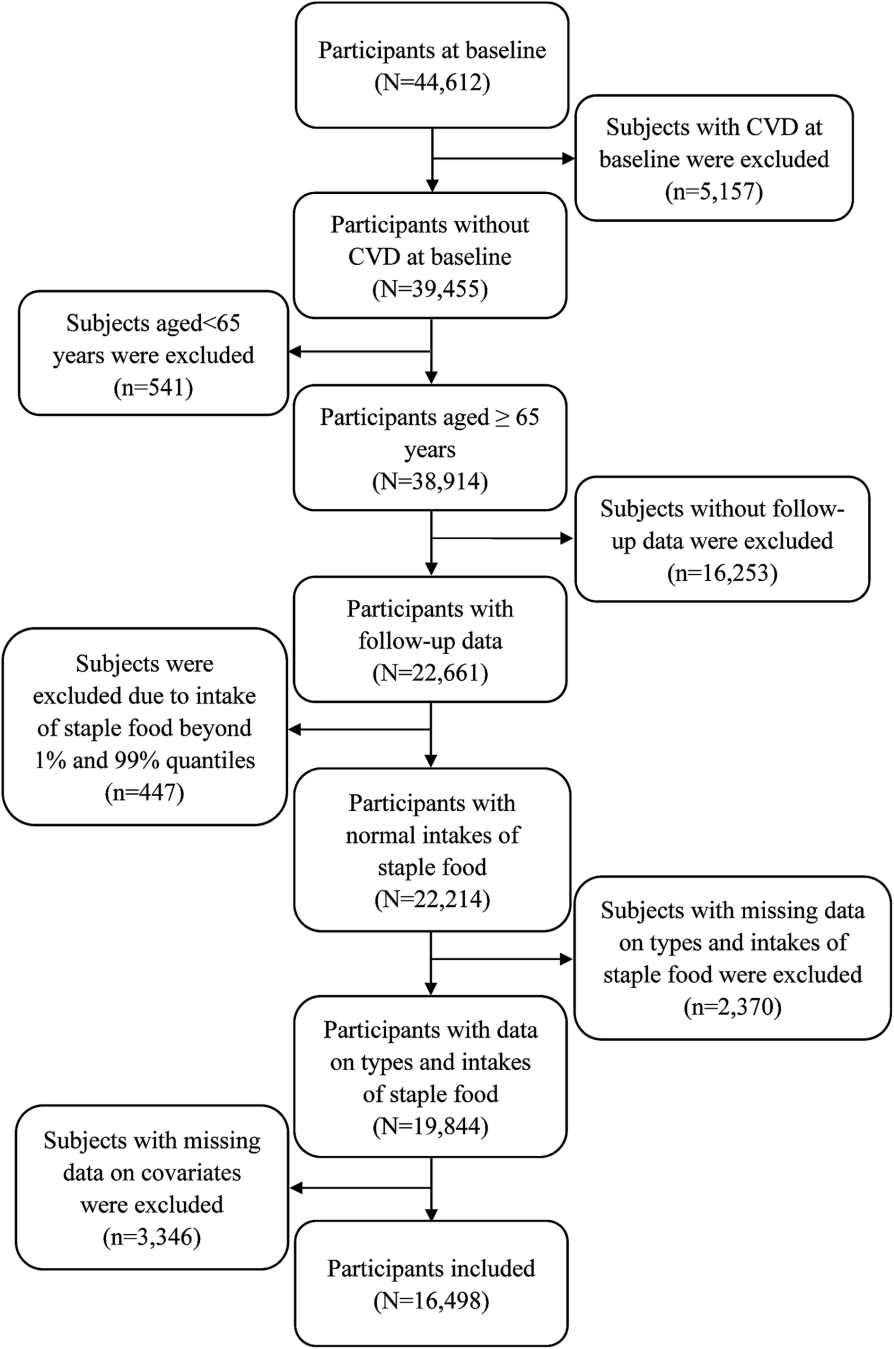
The detailed exclusion of participants.

Participants aged 65 years or older at baseline were eligible for this study. Exclusion criteria included: having CVD at baseline (*n* = 5,157), age under 65 years (*n* = 541), lack of follow-up data (*n* = 16,253), staple food intake outside the 1st and 99th percentiles (*n* = 447), and missing data on staple food types, intake, or covariates (*n* = 5,716). Additionally, participants who first enrolled in the 2018 wave were excluded due to the absence of follow-up data. The final sample comprised 16,498 participants (Figure 1).

Ethical approval was obtained from the Institutional Review Board at Duke University (Pro00062871) and the Biomedical Ethics Committee of Peking University (IRB00001052–13074). Written informed consent was obtained from all participants.

### 2.2 Assessment of staple food types and intake

Staple food types were self-reported using the question, “What is your main grain as a staple food for a long time?” Staple food in the survey included rice, wheat, and coarse cereals. Participants were explained that rice included white rice and rice-based products, wheat included wheat flour and its products, and coarse cereals comprised corn, millet, maize, adlay, buckwheat, oats, purple rice, sorghum, and barley. Intake of staple foods was assessed using the question, “How much grain do you eat on average every day?”

### 2.3 Assessment of CVD

CVD diagnosis was determined through self-reported physician diagnoses obtained during standardized interviews. Participants were asked whether they had ever been informed by a doctor of having conditions such as congestive heart failure, coronary heart disease, angina pectoris, myocardial infarction, or stroke. Participants who reported a CVD diagnosis for the first time after baseline were classified as having an incident CVD event. Follow-up time was calculated from baseline to the occurrence of CVD, loss to follow-up, or death, whichever came first.

### 2.4 Statistical analysis

Continuous variables (e.g., age, body weight, staple food intake) were described as means ± standard deviation (SD) and compared the differences across staple food types using analysis of variance, while categorical variables were expressed as frequencies (percentages) and compared the differences across staple food types using chi-square tests. Cox proportional hazards regression models were employed to calculate hazard ratios (HRs) and 95% confidence intervals (CIs) for associations between staple food types (with rice as the reference) and intake levels (lowest quintile as reference) and CVD risk. The proportional hazards assumption was tested using Schoenfeld residuals. Adjustments were made incrementally: model 1 adjusted for age, sex, body weight, education level, marital status, living area, and ethnicity; model 2 further adjusted for smoking status, alcohol consumption, physical activity, history of hypertension, history of diabetes, self-reported health, and dietary intake of fruits, vegetables, and meat; model 3 additionally adjusted for total staple food intake. Sampling weights were applied to account for non-response bias and oversampling using PROC SURVEYPHREG in SAS 9.4. Stratified analyses were conducted by age (65–79 vs. ≥80 years), sex (male vs. female), and history of hypertension (yes vs. no).

Three sensitivity analyses were performed to ensure robustness: Competing risk Cox models assessed the potential influence of death. Random-effects models, with survey wave as a random term, accounted for staggered participant entry. To reduce reverse causation, participants diagnosed with CVD within 2 years of baseline were excluded.

Restricted cubic splines were used to explore potential non-linear relationships between staple food intake and CVD risk. All statistical analyses were conducted using SAS 9.4 (SAS Institute Inc., Cary, NC, USA). A two-tailed *p* ≤ 0.05 was considered statistically significant.

## 3 Results

### 3.1 Participant characteristics

This study included 16,498 participants with a mean age of 87.57 ± 10.74 years, of whom 42.70% (*n* = 7,045) were male, and 57.30% (*n* = 9,453) were female. Among the participants, 77.12% (*n* = 12,723) reported rice as their primary staple food, 4.81% (*n* = 794) consumed coarse cereals, and 18.07% (*n* = 2,981) consumed wheat. During a median follow-up of 7.38 years, 1757 participants experienced new-onset CVD. Table 1 reveals significant differences across staple food types in all variables except sex (*p* = 0.120), marital status (*p* = 0.401), alcohol consumption (*p* = 0.584), and physical activity (*p* = 0.191).

**Table 1:** Baseline characteristics of all participants.

Characteristics	Total samples (*N* = 16,498)	Types of staple food			
		Rice (*n* = 12,723)	Coarse cereals (*n* = 794)	Wheat (*n* = 2,981)	*p*
Age (years, mean ± SD)^a^	87.57 ± 10.74	87.61 ± 10.73	88.26 ± 11.01	87.23 ± 10.68	0.039
Body weight (kg, mean ± SD)^a^	48.00 ± 10.87	47.17 ± 10.42	49.72 ± 11.85	51.11 ± 11.81	<0.001
Intakes of staple food (g/d, mean ± SD)^a^	309.37 ± 127.75	296.27 ± 121.25	359.19 ± 137.91	352.01 ± 138.95	<0.001
Sex^b^					0.120
Male	7,045 (42.70)	5,396 (42.41)	328 (41.31)	1,321 (44.31)	
Female	9,453 (57.30)	7,327 (57.59)	466 (58.69)	1,660 (55.69)	
Marital status^b^					0.401
Married	4,661 (28.25)	3,559 (27.97)	220 (27.71)	882 (29.59)	
Divorced/ widowed	11,637 (70.54)	9,007 (70.79)	567 (71.41)	2063 (69.20)	
Unmarried	200 (1.21)	157 (1.23)	7 (0.88)	36 (1.21)	
Living areas^b^					<0.001
Urban	6,868 (41.63)	5,543 (43.57)	283 (35.64)	1,042 (34.95)	
Rural	9,630 (58.37)	7,180 (56.43)	511 (64.36)	1939 (65.05)	
Education^b^					<0.001
Illiteracy	10,373 (62.87)	7,773 (61.09)	550 (69.27)	2050 (68.77)	
Primary school or above	6,125 (37.13)	4,950 (38.91)	244 (30.73)	931 (31.23)	
Ethnicity ^b^					<0.001
Han	15,538 (94.18)	11,932 (93.78)	694 (87.41)	2,912 (97.69)	
Others	960 (5.82)	791 (6.22)	100 (12.59)	69 (2.31)	
Smoking status^b^					<0.001
Current	3,206 (19.43)	2,434 (19.13)	142 (17.88)	630 (21.13)	
Former	2,362 (14.32)	1787 (14.05)	94 (11.84)	481 (16.14)	
Never	10,930 (66.25)	8,502 (66.82)	558 (70.28)	1870 (62.73)	
Alcohol consumer status^b^					0.584
Yes	3,676 (22.28)	2,852 (22.42)	166 (20.91)	658 (22.07)	
No	12,822 (77.72)	9,871 (77.58)	628 (79.09)	2,323 (77.93)	
Physical activity^b^					0.191
Current	5,006 (30.34)	3,841 (30.19)	245 (30.86)	920 (30.86)	
Former	1,013 (6.14)	767 (6.03)	40 (5.04)	206 (6.91)	
Never	10,479 (63.52)	8,115 (63.78)	509 (64.11)	1855 (62.23)	
Intake of fruits^b^					<0.001
Always	4,089 (24.78)	3,004 (23.61)	201 (25.31)	884 (29.65)	
Sometimes	8,070 (48.92)	6,247 (49.10)	390 (49.12)	1,433 (48.07)	
Rarely	4,339 (26.30)	3,472 (27.29)	203 (25.57)	664 (22.27)	
Intake of vegetables^b^					<0.001
Always	13,582 (82.33)	10,603 (83.34)	632 (79.60)	2,347 (78.73)	
Sometimes	2,360 (14.30)	1702 (13.38)	142 (17.88)	516 (17.31)	
Rarely	556 (3.37)	418 (3.29)	20 (2.52)	118 (3.96)	
Intake of meat^b^					<0.001
Always	5,469 (33.15)	4,720 (37.10)	137 (17.25)	612 (20.53)	
Sometimes	8,050 (48.79)	5,979 (46.99)	467 (58.82)	1,604 (53.81)	
Rarely	2,979 (18.06)	2024 (15.91)	190 (23.93)	765 (25.66)	
Self-reported health^b^					<0.001
Poor	1,649 (10.00)	1,300 (10.22)	73 (9.19)	276 (9.26)	
Fair	5,468 (33.14)	4,362 (34.28)	269 (33.88)	837 (28.08)	
Good	9,381 (56.86)	7,061 (55.50)	452 (56.93)	1868 (62.66)	
History of hypertension^b^					<0.001
No	6,337 (38.41)	4,968 (39.05)	345 (43.45)	1,024 (34.35)	
Yes	10,161 (61.59)	7,755 (60.95)	449 (56.55)	1957 (65.65)	
History of diabetes^b^					0.001
No	16,277 (98.66)	12,545 (98.60)	775 (97.61)	2,957 (99.19)	
Yes	221 (1.34)	178 (1.40)	19 (2.39)	24 (0.81)	

SD, standard deviation.

a These variables were compared between three groups using analysis of variance.

b These variables were compared between three groups using chi-square test.

### 3.2 Association between staple food types and CVD risk

Table 2 shows that compared to rice, consuming wheat as the primary staple food was related to a 40.8% higher risk of CVD (HR: 1.408; 95% CI: 1.195–1.658; *p* < 0.001). No association of coarse cereals with CVD risk was observed (HR: 1.099; 95% CI: 0.846–1.428; *p* = 0.477). These associations remained consistent after adjusting for lifestyle factors, medical history, and total staple food intake.

**Table 2:** Associations of types of staple food with the incidence of CVD in whole population (*N* = 16,498).

Subgroups	Events/participants	Model 1^a^		Model 2^b^		Model 3^c^	
		HR (95% CI)	*p*	HR (95% CI)	*p*	HR (95% CI)	*p*
Rice	1321/12723	*Ref*		*Ref*		*Ref*	
Wheat	352/2981	1.408 (1.195, 1.658)	<0.001	1.305 (1.103, 1.544)	0.002	1.320 (1.114, 1.564)	0.001
Coarse cereals	84/794	1.099 (0.846, 1.428)	0.477	1.027 (0.788, 1.340)	0.842	1.036 (0.795, 1.351)	0.793

CVD, cardiovascular disease.

a Age, sex, body weight, education levels, marital status, living areas, and ethnicity were adjusted. In stratified analysis, strata factors were not adjusted.

b Age, sex, body weight, education levels, marital status, living areas, ethnicity, smoking status, alcohol consumer status, physical activity, history of hypertension, history of diabetes, self-reported health, and intakes of fruit, vegetables, and meat were adjusted. In stratified analysis, strata factors were not adjusted.

c Age, sex, body weight, education levels, marital status, living areas, ethnicity, smoking status, alcohol consumer status, physical activity, history of hypertension, history of diabetes, self-reported health, intakes of fruit, vegetables, and meat, and intake of staple food were adjusted. In stratified analysis, strata factors were not adjusted.

**Figure 2:**
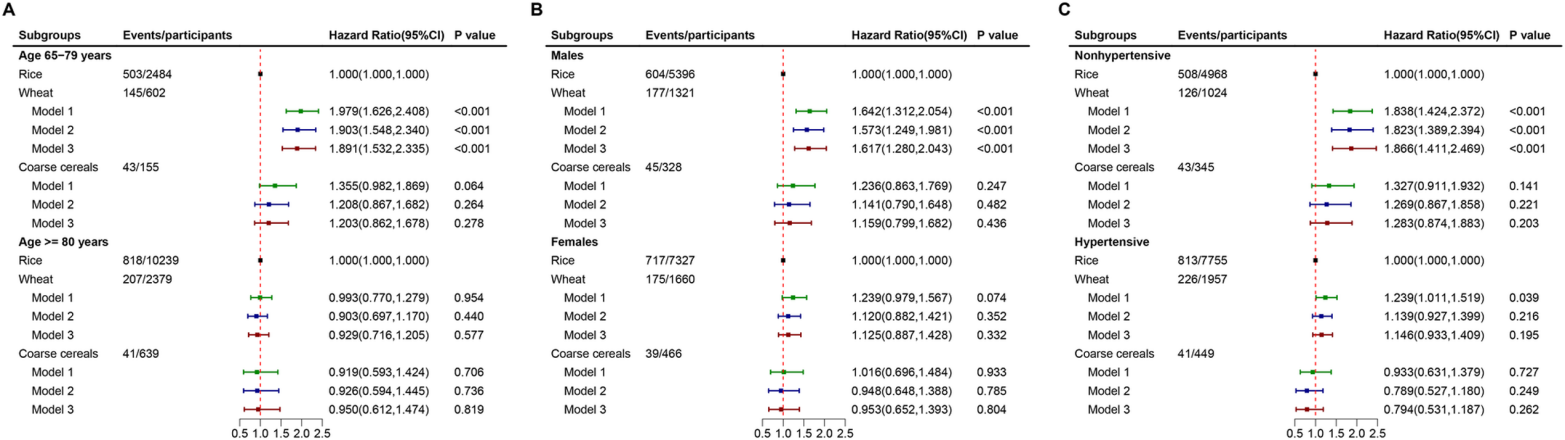
Associations of types of staple food with the risk of CVD stratified by age, sex, and hypertensive status. **(A)**. Stratified by age; **(B)**. Stratified by sex; and **(C)**. Stratified by hypertensive status.

Stratified analyses revealed variations by age, sex, and hypertension history. Among participants aged 65–79 years, wheat consumption was linked to an 89.1% higher CVD risk (HR: 1.891; 95% CI: 1.532–2.335; *p* < 0.001), but no significant associations were observed in participants aged 80 years or older (Figure 2A). For males, wheat consumption was related to a 61.7% higher CVD risk (HR: 1.617; 95% CI: 1.280–2.043; *p* < 0.001), while no significant association was observed in females (Figure 2B). Among participants without hypertension, wheat consumption was linked to an 86.6% higher CVD risk (HR: 1.866; 95% CI: 1.411–2.469; *p* < 0.001), but no association was found among those with hypertension (Figure 2C).

### 3.3 Association between staple food intake and CVD risk

In the overall sample, no significant associations were observed between the intake levels of rice, coarse cereals, or wheat and CVD risk (*p* = 0.204, 0.812, and 0.805, respectively). However, among participants aged 80 years or older, higher rice intake was associated with an 11.3% lower CVD risk per standard deviation increase (HR: 0.887; 95% CI: 0.793–0.991; *p* = 0.035). Stratified analyses by sex and hypertension history showed no significant associations for any staple food type, consistent with the main findings (Table 3).

**Table 3:** Associations of intakes of staple food with the incidence of CVD.

Subgroups	Rice		Coarse cereals		Wheat	
	HR (95% CI)	*p*	HR (95% CI)	*p*	HR (95% CI)	*p*
Total samples (*N* = 16,498)^a^						
Q1	1.000		1.000		1.000	
Q2	0.891 (0.632, 1.257)	0.511	2.219 (0.381, 12.934)	0.375	0.661 (0.256, 1.701)	0.390
Q3	0.923 (0.687, 1.239)	0.594	1.632 (0.279, 9.552)	0.586	0.690 (0.284, 1.678)	0.413
Q4	1.169 (0.891, 1.534)	0.260	1.386 (0.198, 9.676)	0.742	0.480 (0.204, 1.126)	0.091
Q5	0.908 (0.692, 1.191)	0.485	1.435 (0.261, 7.890)	0.678	0.650 (0.289, 1.461)	0.297
Increase per SD	0.953 (0.885, 1.027)	0.204	0.968 (0.744, 1.261)	0.812	0.982 (0.852, 1.133)	0.805
Age 65–79 years (*n* = 3,241)^b^						
Q1	1.000		1.000		1.000	
Q2	1.179 (0.713, 1.951)	0.521	1.324 (0.124, 14.159)	0.815	1.058 (0.262, 4.264)	0.937
Q3	0.931 (0.576, 1.505)	0.771	0.300 (0.022, 4.113)	0.365	0.965 (0.243, 3.830)	0.959
Q4	1.411 (0.916, 2.174)	0.118	0.227 (0.019, 2.780)	0.244	0.549 (0.145, 2.075)	0.376
Q5	1.228 (0.799, 1.887)	0.350	0.158 (0.015, 1.680)	0.125	0.944 (0.260, 3.433)	0.931
Increase per SD	1.023 (0.925, 1.131)	0.661	0.652 (0.411, 1.035)	0.069	1.020 (0.847, 1.227)	0.838
Age ≥ 80 years (*n* = 13,257)^b^						
Q1	1.000		1.000		1.000	
Q2	0.844 (0.532, 1.340)	0.472	9.336 (0.817, 106.727)	0.072	0.320 (0.083, 1.239)	0.099
Q3	1.056 (0.738, 1.509)	0.767	17.599 (0.829, 373.402)	0.066	0.520 (0.173, 1.561)	0.244
Q4	1.147 (0.814, 1.615)	0.432	11.594 (1.872, 71.802)	0.009	0.360 (0.119, 1.089)	0.071
Q5	0.734 (0.505, 1.065)	0.104	47.345 (2.624, 854.134)	0.009	0.412 (0.146, 1.162)	0.094
Increase per SD	0.887 (0.793, 0.991)	0.035	1.727 (1.034, 2.885)	0.037	0.932 (0.727, 1.194)	0.575
Males (*n* = 7,045)^c^						
Q1	1.000		1.000		1.000	
Q2	0.588 (0.340, 1.016)	0.057	0.271 (0.018, 4.005)	0.341	0.576 (0.182, 1.822)	0.347
Q3	0.770 (0.476, 1.244)	0.285	0.036 (0.002, 0.759)	0.033	0.414 (0.149, 1.148)	0.090
Q4	1.144 (0.763, 1.714)	0.515	0.137 (0.008, 2.238)	0.162	0.286 (0.114, 0.723)	0.008
Q5	0.815 (0.545, 1.220)	0.320	0.116 (0.009, 1.570)	0.105	0.337 (0.142, 0.799)	0.014
Increase per SD	0.972 (0.877, 1.077)	0.587	1.042 (0.664, 1.636)	0.858	0.852 (0.710, 1.024)	0.088
Females (*n* = 9,453)^c^						
Q1	1.000		1.000		1.000	
Q2	1.073 (0.707, 1.627)	0.741	4.729 (0.485, 46.082)	0.181	0.600 (0.180, 2.003)	0.406
Q3	1.004 (0.699, 1.443)	0.981	2.794 (0.377, 20.723)	0.314	0.758 (0.247, 2.322)	0.627
Q4	1.203 (0.850, 1.702)	0.298	2.393 (0.281, 20.346)	0.424	0.529 (0.174, 1.609)	0.262
Q5	0.955 (0.670, 1.363)	0.802	2.207 (0.279, 17.442)	0.452	0.815 (0.291, 2.280)	0.696
Increase per SD	0.937 (0.842, 1.042)	0.230	0.919 (0.628, 1.344)	0.662	1.175 (0.935, 1.476)	0.167
Nonhypertensive (*n* = 6,337)^d^						
Q1	1.000		1.000		1.000	
Q2	0.996 (0.585, 1.694)	0.987	2.815 (0.211, 37.635)	0.433	0.317 (0.088, 1.142)	0.079
Q3	0.879 (0.562, 1.376)	0.574	1.254 (0.058, 27.217)	0.885	0.221 (0.080, 0.614)	0.004
Q4	1.054 (0.697, 1.592)	0.803	2.782 (0.145, 53.203)	0.496	0.093 (0.031, 0.274)	<0.001
Q5	0.966 (0.648, 1.441)	0.867	2.128 (0.126, 35.917)	0.600	0.205 (0.084, 0.499)	0.001
Increase per SD	0.964 (0.854, 1.088)	0.554	1.075 (0.658, 1.754)	0.773	0.831 (0.642, 1.076)	0.160
Hypertensive (*n* = 10,161)^d^						
Q1	1.000		1.000		1.000	
Q2	0.873 (0.560, 1.358)	0.546	1.143 (0.254, 5.150)	0.862	1.102 (0.302, 4.027)	0.883
Q3	0.973 (0.664, 1.426)	0.888	1.841 (0.559, 6.059)	0.315	1.429 (0.402, 5.077)	0.581
Q4	1.264 (0.890, 1.796)	0.191	0.942 (0.317, 2.801)	0.914	1.248 (0.388, 4.014)	0.710
Q5	0.874 (0.609, 1.255)	0.466	1.185 (0.314, 4.471)	0.802	1.392 (0.441, 4.396)	0.573
Increase per SD	0.942 (0.856, 1.036)	0.217	1.082 (0.689, 1.700)	0.731	1.055 (0.892, 1.249)	0.531

CVD, cardiovascular disease.

a Age, sex, body weight, education levels, marital status, living areas, ethnicity, smoking status, alcohol consumer status, physical activity, history of hypertension, history of diabetes, self-reported health, and intakes of fruit, vegetables, and meat were adjusted.

b Sex, body weight, education levels, marital status, living areas, ethnicity, smoking status, alcohol consumer status, physical activity, history of hypertension, history of diabetes, self-reported health, and intakes of fruit, vegetables, and meat were adjusted.

c Age, body weight, education levels, marital status, living areas, ethnicity, smoking status, alcohol consumer status, physical activity, history of hypertension, history of diabetes, self-reported health, and intakes of fruit, vegetables, and meat were adjusted.

d Age, sex, body weight, education levels, marital status, living areas, ethnicity, smoking status, alcohol consumer status, physical activity, history of diabetes, self-reported health, and intakes of fruit, vegetables, and meat were adjusted.

### 3.4 Non-linear associations of staple food intake with CVD risk

**Figure 3:**
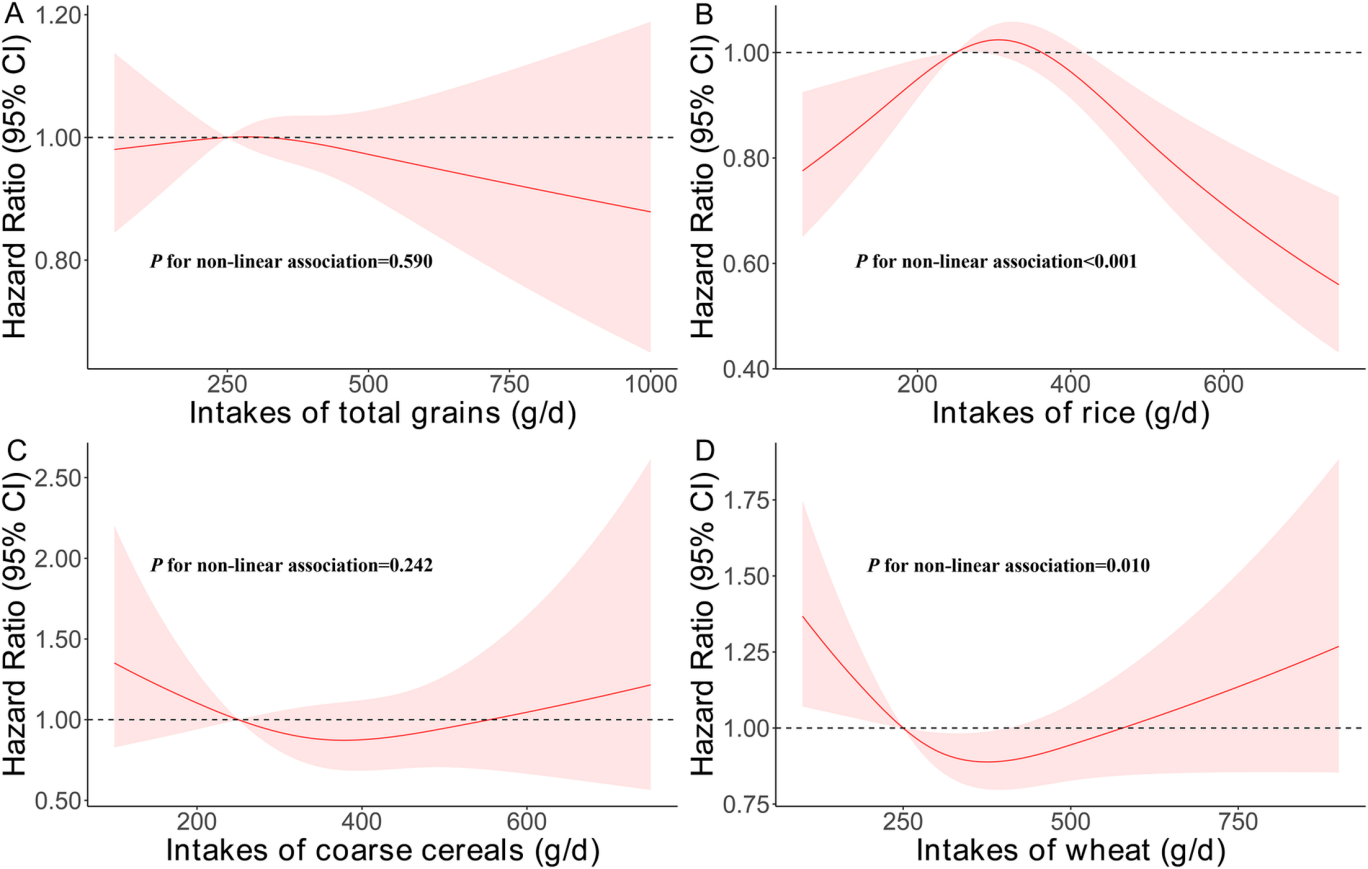
Non-linear associations of intakes of staple food with the risk of CVD. **(A)**. Intakes of rice, wheat, and coarse cereals; **(B)**. Intakes of rice; **(C)**. Intakes of coarse cereals; and **(D)**. Intakes of wheat.

Figure 3 illustrates significant non-linear associations for rice and wheat intake with CVD risk (P for non-linear association<0.001 and *p* = 0.010, respectively), but no significant non-linear relationship was found for coarse cereals. A reverse U-shaped association was observed for rice intake, with both low and high consumption associated with lower CVD risk (Figure 3B). For wheat, a U-shaped relationship was identified, with the lowest CVD risk at a cooked intake level of 375 g/day (Figure 3D).

### 3.5 Sensitivity analysis

The robustness of the results was supported by three sensitivity analyses (Supplementary Table 1). First, a competing risk Cox model accounting for mortality showed similar associations, with wheat linked to higher CVD incidence and no significant relationship between staple food intake levels and CVD risk. Second, analyses incorporating survey wave as a random effect yielded consistent findings. Finally, excluding participants diagnosed with CVD within two years of baseline produced comparable results, confirming the main conclusions.

## 4 Discussion

This cohort study is the first to explore the associations between staple food types, intake levels, and CVD risk among older Chinese adults. Wheat as a primary staple food was linked to an increased risk of CVD compared to rice, particularly among participants aged 65–79 years, males, and those without hypertension. While no linear associations between rice, coarse cereals, and wheat intake and CVD risk were observed, non-linear relationships emerged for rice and wheat consumption.

To our knowledge, this study originally investigated the relationship between staple food types and CVD risk. Our findings indicate that wheat as a staple food may increase the risk of CVD compared to rice. While no direct evidence comparing rice and wheat consumption specifically for CVD risk has been reported, previous research using the CLHLS dataset found that wheat, compared to rice, was positively related to the risk of all-cause mortality, partially supporting our findings (22). The health risks associated with grain consumption may be influenced more by accompanying dietary patterns than the grains themselves (23). For instance, rice is typically consumed alongside soy products and seaweed, which are negatively related to CVD risk and its related risk factors (24–27). Conversely, wheat-based foods, such as bread and noodles, are often paired with red and processed meats, which are positively related to CVD risk (8, 28). Importantly, the significant association between wheat consumption and higher CVD incidence persisted even after adjusting for vegetable, fruit, and meat intake. This suggests that rice may offer intrinsic protective benefits against CVD. Supporting evidence indicates that a bread-and-dairy dietary pattern is positively related to the levels of fasting glucose (29), low density lipoprotein (LDL) cholesterol (30), and lower C-reactive protein (31), while noodle consumption has been linked to elevated cholesterol (30) and phosphorus levels (32). Additionally, rice appears to have favorable nutritional properties, including lower fat and sodium content and the absence of cholesterol (33). Studies have shown that rice consumption is less likely to induce postprandial hyperglycemia and elevated triglyceride levels compared to other carbohydrate sources, such as white bread. These characteristics may help explain rice’s potential protective role in reducing CVD risk (34). Wheat-based products provide approximately 263 kcal and 49 grams of carbohydrates per 100 grams, accounting for about 35% of daily energy intake. In contrast, white rice offers 130 kcal and 28 grams of carbohydrates per 100 grams, contributing roughly 26% to daily energy intake (35). Refined wheat staples generally have a higher glycemic index (GI) than rice. High-GI foods cause rapid increases in blood glucose and insulin levels, which can trigger oxidative stress and chronic hyperinsulinemia. Prolonged exposure to these metabolic effects is associated with insulin resistance—a central component of metabolic syndrome—and with endothelial dysfunction, both of which are established risk factors for CVD. In addition to their high glycemic index, refined wheat products often contain vegetable oils rich in linoleic acid. These oils have been associated with mitochondrial dysfunction and oxidative damage to cardiolipin—a phospholipid essential for energy production in vascular cells. Such cellular damage may impair vascular function and contribute to the development of cardiovascular disease. Previous review has also linked regular consumption of wheat products to chronic inflammation and a heightened risk of autoimmune conditions. Compounds naturally present in wheat, such as gliadin and wheat germ agglutinin, have been shown in both laboratory and human studies to increase intestinal permeability and stimulate immune activation. These effects may further exacerbate systemic inflammation, creating an environment conducive to cardiovascular risk (36).

Age, sex, and hypertension status influenced the associations between staple food types and CVD risk, with significant relationships observed only among participants aged 65–79 years, males, and those without hypertension. First, similar findings have been reported in a prospective study from Japan, which found that rice consumption was negatively related to CVD mortality in males but not in females (37). This may reflect underlying biological differences in CVD development between sexes, including genetic and epigenetic factors, as well as the influence of sex hormones and their receptors (38). Men tend to develop coronary artery disease (CAD) earlier and often exhibit more severe atherosclerosis compared to women. Second, the CLHLS predominantly included the eldest-old adults, who constituted 80% of the cohort. These participants are generally healthier than younger elderly adults, possibly due to genetic resilience. Consequently, lifestyle factors, including diet, may have a diminished role in disease development for this age group compared to genetic influences. Third, the observed association between staple food type and CVD risk was significant only in participants without hypertension. This could be because individuals with hypertension are more likely to adopt healthier lifestyle habits, including dietary changes, to manage their condition, thereby reducing the impact of staple food choices on CVD development. Further research is needed to explore the specific biological and behavioral mechanisms underlying these findings.

This study found no significant linear associations between the intake of rice, wheat, or coarse cereals and the risk of CVD. These findings align with a meta-analysis that reported no association between white rice, total rice, or total grains and all-cause or cause-specific mortality (11). Similarly, a pooled analysis of three U.S. cohorts found no link between white or brown rice consumption and CVD risk (16), and another U.S. study reported no association between rice intake and subclinical CVD markers in a multiethnic population (17). This study collected data on the types and amounts of grains consumed but did not distinguish between refined and whole grain varieties of rice or wheat—an important limitation, given their differing health effects on cardiovascular risk (11, 39). Extensive research indicates that whole grain consumption is associated with a lower risk of cardiovascular disease and reduced overall mortality, while refined grains, including white rice, generally offer no significant protective benefit (11, 23). This distinction is particularly important in regions such as Asia, where white rice remains a dietary staple (40). For instance, studies conducted in Japan and across multinational cohorts in 21 countries have found no significant association between rice intake and cardiovascular disease incidence (35, 41). Given this limitation, the present study was unable to evaluate the distinct effects of refined versus whole grains on cardiovascular outcomes. Interestingly, this study identified non-linear associations between rice and wheat intake and CVD risk. A U-shaped relationship was observed for wheat intake, with the lowest CVD risk at a cooked intake of 375 g/day, consistent with the Chinese Dietary Guidelines (2016) recommendations (42). Previous research has also noted a U-shaped association between rice intake and all-cause mortality but did not observe a similar pattern for wheat. These differences may reflect distinct mechanisms by which rice and wheat impact CVD and mortality risk, warranting further investigation (22).

The CLHLS collected information on grain types and intake only at baseline, assuming that dietary patterns remained stable over time in older adults. However, chronic conditions such as hypertension, diabetes, and cardiovascular disease are common in this population and may lead to changes in dietary habits, including the type and quantity of grains consumed. Although individuals diagnosed with cardiovascular disease within two years of baseline were excluded in sensitivity analyses, the possibility of reverse causality cannot be fully ruled out. Furthermore, the study did not capture detailed dietary information, such as sodium intake, or account for medication use, including antihypertensive or cardiovascular drugs. These unmeasured variables may act as confounders, potentially influencing the observed associations. Given these limitations, the findings should be interpreted with caution and validated through well-designed prospective studies that include comprehensive dietary and clinical data.

### 4.1 Strengths and limitations

The strengths of this study included its prospective design, large sample of older adults, extended follow-up period, and novel insights into the relationship between staple food types and CVD. These features enhance the study’s reliability and relevance for understanding dietary impacts on CVD risk in an aging population. However, there are important limitations to consider. First, the study lacked detailed information on whether participants consumed whole grains or refined grains, preventing a more nuanced analysis of grain type effects. Second, the possibility that some participants consumed both rice and wheat as staple foods could not be fully addressed, as this information was not consistently collected across all waves of the CLHLS. As a result, participants with mixed staple food consumption were excluded from the analysis, which may have influenced the findings. Third, the use of self-reported data to assess grain type and intake may introduce recall bias, particularly among older adults, potentially compromising the accuracy of dietary information. In addition, the study relied on only two basic questions rather than a validated food frequency questionnaire, increasing the likelihood of measurement error in dietary assessment. These factors may have influenced the reliability of the observed associations. Fourth, because height was not recorded until the 2014 wave of the CLHLS, body mass index could not be calculated or included as a covariate, limiting the ability to adjust for this important factor. Finally, the CLHLS cohort focused primarily on the oldest-old population, who may represent a healthier subset of older adults. As a result, caution is warranted when generalizing these findings to younger or more diverse populations. Future research with more comprehensive dietary assessments, anthropometric data, and broader population samples will be essential to confirm and extend these findings.

## 5 Conclusion

This study is among the first to examine the association between staple food types and the risk of CVD in older adults. The findings indicate that, compared to rice, wheat as a staple food is related to a higher incidence of CVD, particularly among younger older adults, males, and individuals without hypertension. However, no significant associations were observed between the intake levels of rice, wheat, or coarse cereals and CVD risk. These findings provide valuable insights for developing targeted dietary guidelines to help prevent CVD in the aging Chinese population. Future research should further explore the underlying mechanisms and broader applicability of these results to refine dietary recommendations for older adults.

## Data Availability

The datasets presented in this study can be found in online repositories. The names of the repository/repositories and accession number(s) can be found at: https://opendata.pku.edu.cn/dataverse/CHADS.
