# Supplementary material for: Relationship between staple food types and cardiovascular disease risk among older Chinese adults
Source: Front Nutr. 2025 May 22;12:1539920. doi: 10.3389/fnut.2025.1539920 (PMC12137065; doi:10.3389/fnut.2025.1539920)
Supplement: Supplementary file 1 [file Table_1.docx]

Supplementary Table 1 Associations of types of staple food and intakes with the incidence of CVD

| Staple food | Model 1 ^a^ | | Model 2 ^b^ | | Model 3 ^c^ | |
| --- | --- | --- | --- | --- | --- | --- |
|  | *HR* (*95% CI*) | *P* | *HR* (*95% CI*) | *P* | *HR* (*95% CI*) | *P* |
| **Types of staple food** |  |  |  |  |  |  |
| Rice | 1.000 |  | 1.000 |  | 1.000 |  |
| Wheat | 1.244(1.146,1.350) | <0.001 | 1.297(1.086,1.548) | 0.004 | 1.322(1.114,1.569) | 0.001 |
| Coarse cereals | 1.054(0.923,1.203) | 0.440 | 1.001(0.765,1.311) | 0.994 | 1.037(0.794,1.354) | 0.791 |
| **Intakes of staple food** |  |  |  |  |  |  |
| Rice | 1.009(0.966,1.054) | 0.680 | 1.011(0.965,1.059) | 0.653 | 0.951(0.882,1.025) | 0.189 |
| Wheat | 1.055(0.897,1.241) | 0.515 | 1.129(0.972,1.311) | 0.111 | 0.968(0.742,1.264) | 0.813 |
| Coarse cereals | 1.018(0.945,1.097) | 0.631 | 0.982(0.907,1.063) | 0.655 | 0.977(0.845,1.128) | 0.747 |

CVD: cardiovascular disease

^a^ A competing risk Cox proportional hazard regression models was used to examine the associations of types and intakes of staple food with the incidence of CVD to assess the influence of death.

^b^ Wave where participants entered the cohort study was adjusted.

^c^ Participants firstly diagnosed with CVD within two years after baseline were removed (n=16467).
